# Airborne protein concentration: a key metric for type 1 allergy risk assessment—in home measurement challenges and considerations

**DOI:** 10.1186/s13601-018-0196-9

**Published:** 2018-03-26

**Authors:** Liz Tulum, Zoë Deag, Matthew Brown, Annette Furniss, Lynn Meech, Anja Lalljie, Stella Cochrane

**Affiliations:** SEAC Unilever Colworth, Colworth Science Park, Sharnbrook, Bedfordshire MK44 1LQ UK

**Keywords:** Indoor aeroallergens, Dust, Air sampling, Respiratory allergy, Protein risk assessment

## Abstract

**Background:**

Exposure to airborne proteins can be associated with the development of immediate, IgE-mediated respiratory allergies, with genetic, epigenetic and environmental factors also playing a role in determining the likelihood that sensitisation will be induced. The main objective of this study was to determine whether airborne concentrations of selected common aeroallergens could be quantified in the air of homes using easily deployable, commercially available equipment and analytical methods, at low levels relevant to risk assessment of the potential to develop respiratory allergies. Additionally, air and dust sampling were compared and the influence of factors such as different filter types on allergen quantification explored.

**Methods:**

Low volume air sampling pumps and DUSTREAM^®^ dust samplers were used to sample 20 homes and allergen levels were quantified using a MARIA^®^ immunoassay.

**Results:**

It proved possible to detect a range of common aeroallergens in the home with sufficient sensitivity to quantify airborne concentrations in ranges relevant to risk assessment (Limits of Detection of 0.005–0.03 ng/m^3^). The methodology discriminates between homes related to pet ownership and there were clear advantages to sampling air over dust which are described in this paper. Furthermore, in an adsorption–extraction study, PTFE (polytetrafluoroethylene) filters gave higher and more consistent recovery values than glass fibre (grade A) filters for the range of aeroallergens studied.

**Conclusions:**

Very low airborne concentrations of allergenic proteins in home settings can be successfully quantified using commercially available pumps and immunoassays. Considering the greater relevance of air sampling to human exposure of the respiratory tract and its other advantages, wider use of standardised, sensitive techniques to measure low airborne protein concentrations and how they influence development of allergic sensitisation and symptoms could accelerate our understanding of human dose–response relationships and refine our knowledge of thresholds of allergic sensitisation and elicitation via the respiratory tract.

## Background

Exposure to airborne allergenic proteins can be associated with the development of immediate, IgE-mediated respiratory allergies such as hay fever or baker’s asthma The focus of this paper is measurement of exposure and as such other risk factors (genetic, epigenetic and environmental) associated with development of allergic sensitisation are not covered in detail here. It is important to appreciate however that not all individuals exposed to airborne allergenic proteins, but rather a subset, are at risk of sensitisation. Whilst measurement of airborne protein concentrations has been widely undertaken in many occupational settings, for example wheat flour proteins in bakeries, to understand potential risks to worker health [1], this approach is less frequently applied in other settings. Thresholds of allergic sensitisation and elicitation to proteins via respiratory exposure remain poorly defined. A few studies have included measurement of airborne protein concentrations during use of products by consumers. Results have been used to define exposure benchmarks for consumer risk assessment [2–8], but the number of these studies in the published literature remains limited. In contrast, the most commonly used technique in studies of environmental allergen exposure in homes and schools is still measurement of allergen in dust samples [e.g. 9–12] as a ‘surrogate’ indicator of exposure. As recently highlighted by Custovic (2015), whilst exposure to allergen(s) is a prerequisite for sensitisation, we do not fully understand human dose–response relationships with regards to allergen exposure and subsequent sensitisation and/or elicitation [13]. Custovic 2015 called for the development of standardised, reliable and reproducible methods for measuring allergen exposure. To this suggestion could be added the need for a standardised, relevant metric, which we propose should be inhalable protein concentration, as is used in existing risk assessment and management approaches.

This paper describes work undertaken as a proof of principle study, to investigate the feasibility of using commercially available air sampling pumps, deployable by study participants, and allergen measurement techniques to quantify airborne protein concentrations (in this case of common environmental aeroallergens) in homes, at concentrations relevant to potential use in risk assessment and furthering our understanding of human-dose response relationships associated with the development of IgE mediated allergies.

The main objective of the study was to determine whether 11 common aeroallergens could be quantified, down to sub ng/m^3^ concentrations, in the air of 20 homes using a low volume air sampler. Secondary objectives were to understand the challenges associated with taking such measurements, investigate the influences of different factors such as filter material on such measurements and establish general feasibility and acceptability in a domestic environment.

## Methods

### Demographics

The occupants of 20 homes in Bedfordshire (United Kingdom) and the surrounding counties agreed to take part (minimum inhabitants in home n = 2). Data was collected from urban and rural areas—50:50 distribution. Sampling occurred during 2 weeks in December 2014 (winter) in the UK in the lounge and a bedroom in each home.

Information about the environment in the lounge and bedroom used for sampling were collected through a questionnaire. The questionnaire requested each participant to provide information on their general cleaning routine, details about the layout of the lounge and bedroom, how often bed linen was changed and how often vacuuming was carried out. Information about the number of people in the household and details about soft furnishings in the rooms was also requested as part of the questionnaire.

The number of people and pets in the house and/or room being monitored and the proportion of time they spent in the room during the lounge run were recorded by questionnaire. The typical activities of the occupants of the room were also recorded during this run. At least one member of the household was at home all day during this study, as they needed to be present to do the sampling and all participants were asked to go about their daily activities in both rooms in which samples were taken. As stated they were asked to hoover the lounge and change the bed to release potential reservoirs of allergen into the air but they could also have been present in either room throughout the sampling period.

Some panellists recorded that they brought their Christmas trees and decorations down from their lofts at the time of the study, which could have introduced additional dust or airborne allergens into the indoor environment.

Participants were requested not to clean extensively the weekend before the study.

All subjects provided written Panellist Agreement and retained a copy of the Experimental Design and Procedures. An ethical review was not required as the study did not involve any intrusive procedures, sampling of human tissues, nor capturing of identifiable, personal data.

### Sampling and assays

#### Indoor air sampling

Subjects collected two air samples on two separate days using an air sampling pump (Casella TUFF™ 4 + , Bedford, UK, 3.5 l/min), connected to an IOM (Institute of Occupational Medicine, SKC Inc. PA) sampling head, which is a simple filter holder. During the day the pump was located either in the lounge or the bedroom for a 10-h sampling period (thus sampling a total air volume of 2.1 m^3^) in each location. Samples were collected onto glass fibre filters (grade A) (Casella Measurement; 25 mm diameter, 0.8 µm pore size) the most commonly used and typically recommended filter for the pumps. During the bedroom sampling, the bed linen was changed 30 min into the sampling period. At the end of the study, the filters were refrigerated until they could be dispatched to Indoor Biotechnologies Ltd for analysis.

Details of the sampling sites were recorded in a questionnaire. The sampling head attached to the TUFF 4 PLUS AIR SAMPLER was situated in the lounge approximately 1 m above floor level, attached to a retort stand and clamp. The pump itself could be placed on the carpeted floor to dampen the noise from the device, if required.

In the bedroom, the IOM sampling head attached to the TUFF 4 PLUS AIR SAMPLER was situated in the bedroom on a bedside table at pillow height. If the room did not contain a bedside table the sampling head was clamped at approximately 1 m from the floor. The pump itself could be placed on the carpeted floor to dampen the noise from the device.

Participants were advised that ‘the pump should not be situated near to the television due to possible electrostatic disturbance’.

### Indoor dust sampling

A sample of dust from the flooring of each lounge was also collected by vacuuming two A4 areas 30 min into the air sampling time using a DUSTREAM^®^ Collector containing nylon collection filters (pore size 40 µm) (Indoor Biotechnologies Ltd, Cardiff) [6].

### MARIA^®^ assay analysis

The GF/A filters and dust samples from the in home study were placed into separate centrifuge tubes and extracted using PBS-Tween (0.05%); 2 ml of PBS-Tween was added to each air filter and per 100 mg of dust. Each centrifuge tube was then placed in a rocker for 2 h at room temperature. The extracts were then analysed by Indoor Biotechnologies Ltd using their fluorescent **M**ultiplex **AR**ray for **I**ndoor **A**llergens (MARIA^®^) technology (Indoor Biotechnologies Ltd, Cardiff, UK) [14, 15]. The MARIA^®^ technology is based on xMAP^®^ technology (Luminex Corp. Austin TX) which uses polystyrene microspheres that are labelled to create distinct sets of microspheres. Separate bead sets are covalently coupled with allergen-specific monoclonal antibodies, enabling the simultaneous capture and detection of multiple allergens in a single sample. The MARIA^®^ assay allowed simultaneous detection of: mite (Der p 1, Der f 1, Mite Group 2), cat (Fel d 1), dog (Can f 1), mouse (Mus m1), rat (Rat n 1), German Cockroach (Bla g 2), Birch pollen (Bet v 1), Alternaria rot fungus (Alt a 1) and peanut allergens (Ara h 6—for dust and air samples this assay was in process of validation, however for filter comparison it had been validated).

### Filter comparison

Separate and in parallel to the in-home study, allergen adsorption and extraction from two different filter types (37 mm GF/A and 47 mm PTFE filters) was compared to investigate how filter type could impact upon allergen quantification. To achieve this, solutions of purified allergens, containing the 11 allergens used in the MARIA assay, were prepared in PBS (phosphate buffered saline, pH 7.4). 75 µl of these solutions were then added to 2 ml of PBS-Tween (0.05% Tween) in separate tubes such that each tube contained a total of 1, 5 and 10 ng of each allergen. Clean PTFE or GF/A filters were then inserted into the tubes and placed on a rocker for 2 h. These ‘adsorption–extraction’ samples were prepared in triplicate. ‘No filter’ control samples were also prepared by simply omitting addition of a filter. Control solutions were also analysed immediately after preparation and before rotation, the results of this analysis (not shown) were not significantly different from the rotated solutions. Allergen concentrations in the solutions after 2 h on a rocker were determined by Indoor Biotechnologies Ltd using the MARIA^®^ assay as described in the preceding text.

## Results

### Filter comparison

**Fig. 1:**
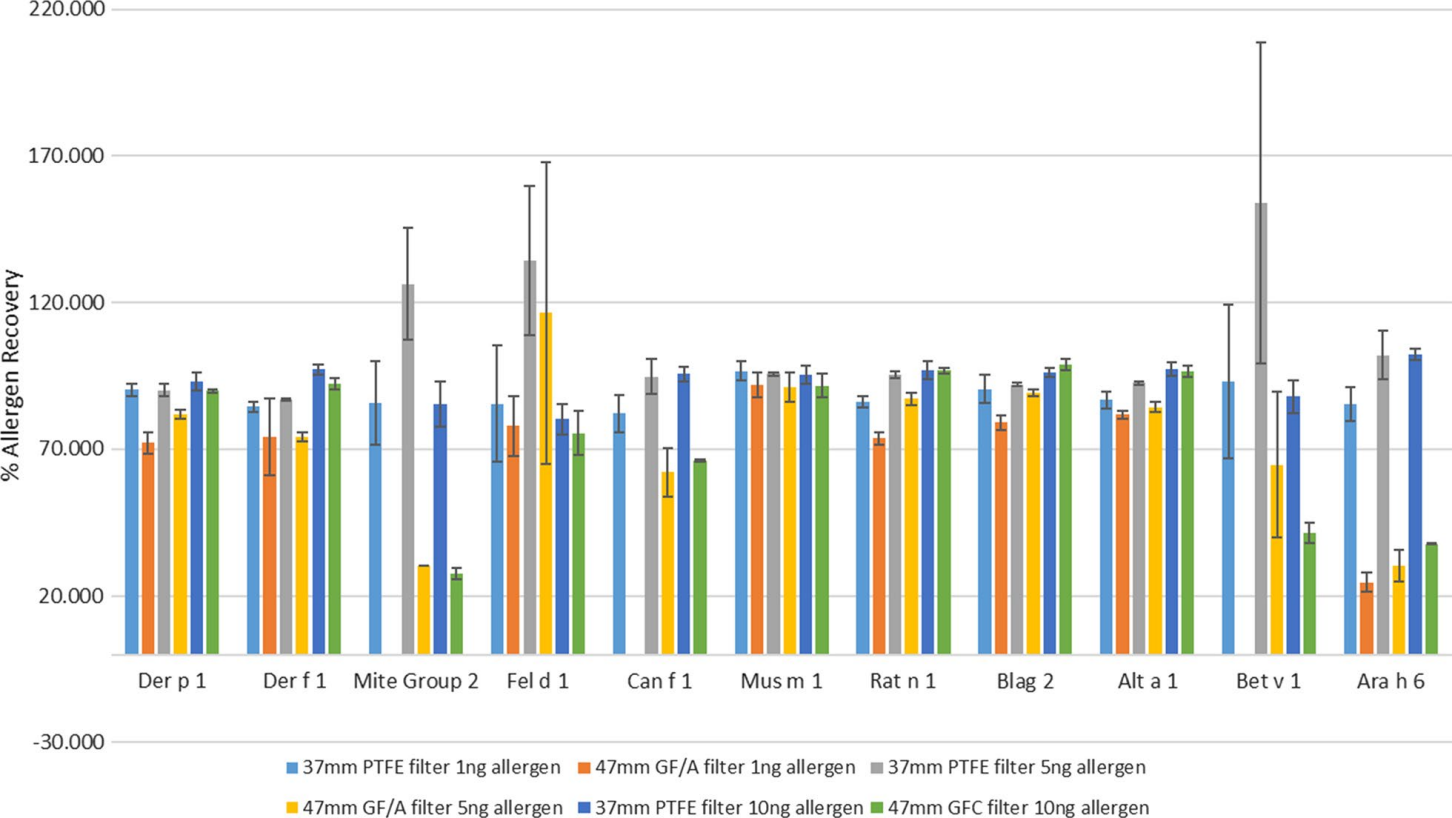
Percentage allergen recoveries (versus the no filter control samples)—a comparison of PTFE and GF/A filters incubated with 1, 5 or 10 ng allergen for 2 h. Average values with standard deviations

All samples were analysed at three dilutions: neat, 1:2 and 1:4. A high degree of reproducibility was observed between different dilutions with an average CV (coefficient of variation) of 5.06%. The average variability between replicates was 7.16% (data not shown). The amounts of allergen measured using the MARIA^®^ assay are summarised in Fig. 1 for the two types of filters incubated with 1, 5 and 10 ng allergen respectively.

The results indicate that the PTFE and GF/A filters absorb and release allergens to different extents, which also varies depending on the allergen and the concentration of the allergen present in the solution. For the majority of allergens (7/11) there was little difference between filter types or PTFE gave a slightly better recovery and equivalent recovery across the allergen doses e.g. as for mus m1. For four allergens however GF/A filters gave poor recovery values compared to PTFE and for 3 allergens (mite group 2 allergens, Can f 1 and Bet v 1) with no recovery at the lowest allergen dose which then plateaus significantly below the PTFE values at the higher doses. For the 4th allergen—Ara h 6—there was no difference in recovery across all doses, although recovery from the GF/A filters was lower, as already noted.

### In home study: demographics and study participant observations

The minimum and maximum number of inhabitants were 2 and 4 respectively. 7/20 homes had 2 inhabitants, 6/20 3 and 7/20 4.

Six households had a cat (one cat) and six had a dog (one which had two), none had both. One household had two guinea pigs and a cornsnake (fed mice).

Hoovering frequency was mostly weekly (12/20 homes) with the maximum and minimum frequencies being daily and every 2 weeks respectively. The duration of hoovering undertaken during the study ranged from 4 to 28 min.

The most typical frequency for bed linen changing was once every 2 weeks (13/20 homes) with the maximum and minimum frequencies being weekly and monthly respectively. The duration taken to change bed linen during the study was reported to be 6–20 min.

11 (55%) homes had carpet in the lounge versus 9 with laminate flooring and 18 (90%) had carpet in the bedroom versus 2 with laminate flooring.

Peanuts were consumed regularly in 8 of the 20 homes.

The majority of panellists found the sampling protocols easy to follow, however, there were a few instances of pumps either not starting properly, or (more rarely) switching themselves off mid-run, indicating that the TUFF 4 air sampler internal timers can sometimes be unreliable, and that use should be carefully monitored and real-time study support provided for panellists to address and minimise the impact of such events upon any sampling (as was the case for this study). The pumps were relatively noisy to run but it was found that the noise could be dampened easily by placing the pumps on a carpeted surface. Panellists did quickly habituate to the noise throughout the 10-h run. It is possible to buy or make noise-cancelling boxes. In light of these findings the authors briefly investigated use of an alternative commercially available sampling pump, the SKC Leland Legacy, with a higher sampling rate of approximately 12 l/min. This was successfully used with a shorter (7 h) sampling period (data not shown).

### In home measurements: general observations

Common aeroallergens detected in the study were as follows: Der p1, Der f1 and mite group 2, cat, dog, rat, mouse, birch pollen, peanut (Ara h6). Mould (Alt a 1) and cockroach (Bla g 2) allergen were below the limit of detection (LoD) in all homes. Table 1 provides an overview of the maximum and minimum values recorded for each allergen across the homes, where they were detectable, along with the LoD. Overall allergen concentration ranges were 0.003–245 µg/g for dust and 0.005–18 ng/m^3^ in air. The limit of detection ranges for the allergens were 0.002–0.012 µg/g for dust and 0.005–0.03 ng/m^3^ for air. These are extremely low detection ranges when compared to those achievable with conventional ELISA results.

**Table 1:** Minimum and maximum concentrations for all allergens measured above the LoD in air (lounge and bedroom) and dust (lounge only, where there was sufficient sample for extraction) across 20 homes

Sample location	Der p 1	Der f 1	Mite group 2	Fel d 1	Can f 1	Mus m 1	Rat n 1	Bet v 1	Ara h 6
Allergen concentration in ng/m^3^ air									
LOD ng/m^3^ air	0.03	0.03	0.01	0.01	0.03	0.005	0.01	0.02	0.01
Lounge air min	0.05	ND	ND	0.2	0.07	ND	ND	ND	ND
Lounge air max	0.16	ND	0.04	14.1	9.74	ND	ND	ND	0.04
Bedroom air min	ND	ND	ND	0.13	ND	ND	ND	ND	ND
Bedroom air max	0.28	0.04	0.06	17.9	2.76	0.005	ND	ND	ND
Allergen concentration in µg/g dust									
LOD µg/g dust	0.012	0.012	0.004	0.004	0.012	0.002	0.004	0.01	0.004
Lounge dust min	0.014	0.028	0.009	0.006	0.014	0.003	0.02	0.02	0.005
Lounge dust max	17.80	9.042	10.010	42.01	244.89	0.005	0.06	0.07	3.73

*LOD* limit of detection, *ND* not detectable above LOD in any of the homes

As described in the filter comparison section GF/A filters, which were used for air sampling in the homes, gave poor recovery values compared to PTFE for 4 allergens (mite group 2 allergens, Can f 1, Bet v 1 and Ara h 6). Therefore, due to the uncertainty associated with the exposures measured for these allergens the absolute values in Table 1 should be treated with caution and whilst some observations associated with their detection are included in the following text, they were omitted from any statistical analyses.

A descriptive narrative is provided in the following text to give insights into the techniques used and where statistical analyses were possible the results are indicated in the text and relevant figure legends.

### Lounge (air and dust measured)

If a home was cat or dog-free no corresponding allergens were detected in the air of the lounge however, specific allergen (Fel d 1 and/or Can f 1) was detected in the homes of people with either a cat or a dog. For example, for Fel d 1 airborne concentrations in lounges ranged from 0.24 to 14.1 ng/m^3^ (mean 3.25 ng/m^3^) in the 6/20 homes with cats, whereas no airborne Fel d 1 was measurable above the LoD in the 14/20 homes without cats. In contrast, dust levels did not reflect ownership—Fel d 1 was detected in dust even when animals were not present in the home, although there was a significant difference (P < 0.001) in the amount detected in homes with cats compared to those without when the data each group was compared by *T* test (unpaired, 2-tailed). Fel d 1 dust levels in lounges from homes with cats ranged from 10.5 to 42.01 µg/g compared to < LoD-0.168 µg/g in homes without cats.

**Fig. 2:**
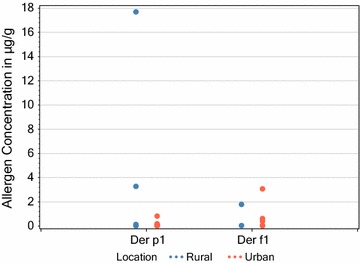
Comparison of measurable (above the LoD) mite allergen dust concentrations in the lounges of rural and urban homes (respective rural and urban sample sizes (n) were 5 and 8 for Der p1 and 2 and 5 for Der f1). Statistical analysis (T-test unpaired 2 t-tailed) comparing rural and urban measurable values gave P-values of 0.157 and 0.779 for Der p 1 and Der f 1 respectively

**Fig. 3:**
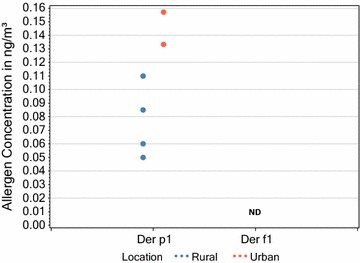
Comparison of measurable (above the LoD) mite allergen airborne concentrations in the lounges of rural and urban homes (Respective rural and urban sample sizes (n) were 4 and 2 for Der p1). Statistical analysis (T-test unpaired 2 t-tailed) comparing rural and urban measurable values P = 0.042. ND = not detected above the LOD

There was no significant difference in the levels of mite allergens (Der p 1 and Der f 1) found in rural homes when sampling dust, but some households didn’t have enough dust sample for analysis. In contrast, the airborne concentrations of Der p 1 were significantly higher in urban homes as shown in Figs. 2 and 3.

**Fig. 4:**
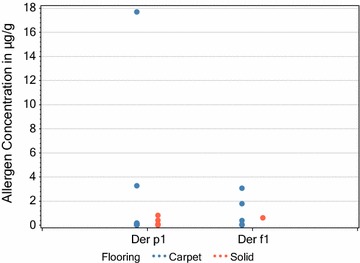
Comparison of measurable (above the LoD) mite allergen dust concentrations in lounges with carpets and those with solid flooring (Respective carpet and solid flooring sample sizes (n) were 9 and 4 for Der p1 and 6 and 1 for Der f1). Statistical analysis (T-test unpaired 2 t-tailed) comparing carpet and solid flooring measurable values gave a P-values of 0.569 for Der p 1. Analysis was not possible for Der f 1 due to the single solid flooring value

**Fig. 5:**
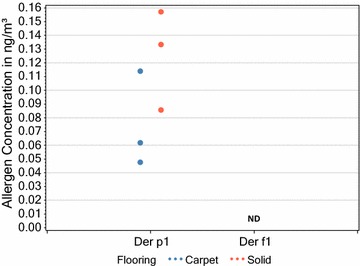
Comparison of measurable (above the LoD) mite allergen airborne concentrations in lounges with carpets and those with solid flooring (Respective carpet and solid flooring sample sizes (n) were 3 and 3 for Der p1) Statistical analysis (T-test unpaired 2 t-tailed) comparing carpet and solid flooring measurable values gave a P-value of 0.152 for Der p 1. ND = not detected above the LOD

When comparing homes containing carpet with those with solid flooring. As shown in Figs. 4 and 5 there were no significant differences in the concentrations of the 2 mite allergens in dust in homes with carpet compared to those with solid flooring, as was also the case when air was sampled. However, some homes, containing both carpet and solid flooring, yielded insufficient dust during sampling to give accurate results.

Other aeroallergens found at detectable levels in the lounge air of some homes were: Ara h6 and Bet v 1, despite being 2 of the allergens more difficult to extract from GF/A filters. It was interesting to note that birch allergen was detectable in the air of the lounge of 2 homes in December, one of these was in a rural location with a birch tree in extremely close proximity.

### Bedroom (only air measured)

Ara h 6 was measurable in the bedroom air of only one home, but again this may reflect the difficulties associated with extracting this allergens from GF/A filters. Other aeroallergens found at detectable levels in the bedroom air of some homes were Der p1, Der f 1, Mite group 2, Fel d 1, mus m1 and Can f 1.

## Discussion

We demonstrate in this pilot study that it is possible to quantify very low airborne concentrations of a range of common aeroallergens, in homes using a commercially available Tuff 4 Plus pump sampling at low volumes (3.5 l/min) over long periods of time (10 h) coupled with analysis of GF/A filters using a MARIA^®^ immunoassay.

The method used had enough sensitivity to quantify very low levels of allergen (0.005–18 ng/m^3^), encompassing values relevant to the risk assessment of exposure to proteins through use of consumer products (0.1–15 ng/m^3^) [1, 6]. It could also be of use in investigating and refining our understanding of human-dose response relationships and thresholds of allergic sensitisation and/or elicitation. However care should be taken when comparing data and benchmarking exposure values between studies to ensure all variables and potential confounding factors are considered, for example the use of high or low air volume sampling pumps and different filter types etc.

In terms of the filters used for detection of aeroallergens the data from the adsorption–extraction study suggests PTFE filters should be the preferred choice in the absence of any further information. However only a small number of allergens have been studied and validation studies should be undertaken to confirm the optimum filter type for allergen capture and subsequent extraction, which should optimized to prevent absorption to glassware and filter e.g. inclusion of Tween 20. As GF/A filters were used for the air sampling in the in home study the poor recovery noted for GF/A versus PTFE filters for mite group 2 allergens, Can f 1, Bet v 1 and Ara h 6 are such that we cannot rule out absorption to the filter as a confounding factor impacting upon detection and quantification of these allergens.

The study also showed clear advantages to air sampling compared to dust sampling. In some situations, there was simply insufficient (house) dust for extraction, whereas air can always be sampled. Additionally, the manner in which dust accumulates appears to blur the relationship between allergen levels in dust and relevant household characteristics such as pet ownership. Of course, it is air that is inhaled, not settled dust, and therefore an airborne concentration is most relevant to human respiratory tract exposure in a risk assessment context. Airborne measurements can provide insights into the patterns of exposure whereas, at best, dust allergen content is more akin to a proxy for cumulative exposure.

Relationships were observed between homes in which peanut is eaten, the ownership of a cat/dog, pet free homes, and the presence of the applicable allergen in both air and dust.

Despite the reduced extraction efficiency observed with Ara h 6 and GF/A filters the peanut allergen Ara h 6 was detected in both air and dust samples from the lounges of 2 households and in the air sampled in the bedroom of one of these households. This is in contrast to the findings of Brough et al. [9] who failed to detect peanut in air samples from homes with high peanut levels in dust. Brough et al. used the same air sampling device as in this study and GF/A filters, but did not detect peanut allergen despite sampling for longer (22 h). However the limit of quantification of their analytical method was equivalent to 2.5 µg/m^3^, compared to a limit of detection in this study of 0.01 ng/m^3^, i.e. 5 orders of magnitude higher. Additionally, the assay used by Brough et al. [9], was the Neogen Veratox^®^ peanut ELISA, designed primarily for use with foods, which according to Jayasena et al. [16] is most sensitive in the recognition of peanut allergens Ara h 3 and Ara h 1 and least sensitive in recognizing the more stable peanut allergens Ara h 2 and Ara h 6 [17]. Differences in stability may influence persistence in the home and therefore assays targeting a more stable allergenic protein could provide insight into exposure to allergenic proteins with the potential to persist and accumulate. Brough et al. [9] concluded ‘Thus peanut protein is unlikely to cause either peanut sensitization or allergic manifestations in patients with peanut allergy through inhalation unless the peanuts are deshelled in close proximity to them’ a statement which cannot be substantiated in the light of our results. Birch pollen in air in December also suggests that in-home ‘build up’ can result in year-round exposure to allergens typically considered seasonal.

If homes were cat-free this was reflected by the lack of detectable Fel d 1 in air samples. However when dust was sampled Fel d 1 was detectable in almost all cat-free homes (though at lower levels than in homes with cats) illustrating that dust, as mentioned, is more akin to a proxy for cumulative exposure as opposed to air, which has the potential to provide data on patterns of exposure. Whilst absolute Fel d 1 values in air and dust samples were lower than reported in previous studies, reflecting the increased sensitivity (typically 10 × more sensitive) of the approach used and other variables such as a small number of homes, the magnitude of difference (2–3 orders of magnitude for dust) between cat-owning and cat-free homes was similar. For example, Custovic et al. [18] sampled dust from 75 British homes using a very similar approach, though coupled with a less sensitive immunoassay and reported an average Fel d 1 dust concentration of 237 µg/g in living rooms of homes with cats, whereas in this study the average concentration in lounge dust was 20 µg/g. In homes without cats, levels were ~ 260 × lower (mean of 0.9 µg/g) in the Custovic et al. study compared to ~ 500 × lower (mean of 0.04 µg/g) in this study. Custovic et al. [18] reported airborne Fel d 1 concentrations of 0.7–38 ng/m^3^ in homes with cats and 0.24–1.78 ng/m^3^ without cats (detectable in 22/75 i.e. 30% of such homes) compared to 0.24–14.1 ng/m^3^ in the 6/20 homes with cats in our study and no detectable airborne Fel d 1 in any of the 14 homes without cats.

Using the same sampling pump as in this study but sampling variable volumes in *bedrooms* Custovic et al. [18] reported Fel d 1 airborne concentrations were 0.4–28 ng/m^3^ in homes with cats and < 0.4 ng/m^3^ in homes without cats. In comparison, in our study Fel d 1 airborne concentrations were 0.13–17.87 ng/m^3^ in bedrooms of homes with cats and < 0.01 ng/m^3^ in those without cats.

Custovic et al. [19] also investigated the relationship between dust and air measurements for Der p 1 and Can f 1 in addition to Fel d 1. Again using similar dust sampling, but for air sampling a higher volume air sampler (60 l/min) was used. Airborne Der p 1 was below the LoD of the assay used i.e. < 0.8 ng/m^3^ in all homes, with Der p 1 levels in living room carpet dust ranging from 0.2 to 66 µg/g (mean 1.14 µg/g). In contrast, in our study, in lounges, airborne Der p 1 levels were detectable (> 0.03 ng/m^3^) and measurable in 6 out of 20 homes. This illustrates that despite using a lower volume air sampler, when coupled with a sensitive immunoassay (MARIA^®^) airborne concentrations of Der p 1 could be quantified in homes with apparently lower Der p 1 dust reservoirs. Can f 1 data were not compared due to the aforementioned potential confounding factor associated with the use of GF/A filters and Can f 1 measurement in this study.

Custovic et al. [19] recognised the need for standardised methods to measure allergen exposure to enable robust assessment of the relationship between exposure, sensitisation and allergic symptoms. At the time of publication, it was concluded that the aerodynamics of each allergen should be considered and for ‘larger/heavier’ allergens such as dust mite allergens measuring levels in dust appeared to be best available index of exposure. However, whilst an index of exposure may be useful in understanding clinically relevant exposures for individuals, in order to further our understanding of the quantitative relationship between exposure and risk of sensitisation or development of allergic symptoms the quantity of inhaled allergen would be the ideal measure of personal exposure. We echo these thoughts and show in this publication that it is technically feasible with readily available and easy to use equipment and assays.

One aspect of airborne allergen exposure that is still poorly understood is the role of high peak airborne concentration exposure versus chronic low airborne exposure on sensitisation or elicitation. More extensive use of ‘personal’ exposure monitoring could provide insight regarding this issue. The pumps used in this study have successfully been used in a ‘personal’ monitoring study, with modifications so they could be carried and sampling timed over 24 h coupled with recording of participants’ location and activity [20, 21]. One could envisage a similar study also incorporating a symptom diary to understand the relationship between exposure to an allergen and elicitation of symptoms in individuals with symptomatic allergy to that allergen.

In summary, whilst there have been some studies using air sampling to understand allergen exposure, the use of different combinations of high and low volume pumps sampling variable amounts of air, coupled with relatively insensitive immunoassays has led to variable success and a perception of the measurement of the allergen concentration of air being technically challenging and requiring specialist equipment. By contrast dust sampling is perceived as simpler and therefore still dominates as the technique used to obtain some ‘index’ of allergen exposure. This situation has hindered progress in our understanding of human dose–response relationships with regards to the development of immediate IgE mediated allergy to airborne proteins, which are essential to quantitative risk assessment. Now, with a variety of pumps sampling at different rates being easily available and adaptable, improved protein capture filter materials and improvement in allergen detection and characterisation (e.g. stability and potential for persistence and home build up) we propose that it is time for a change and hopefully a step forward in research into human inhalational exposure to proteins. Finally, we would also suggest it is time to study exposure to a wider range of proteins, including those not associated with respiratory allergy, to further understand differences in allergenic ‘potency’.

## Conclusions

Our study demonstrates that in-home measurements of low airborne allergen concentrations, at levels relevant for risk assessment of potential allergic sensitisation, are possible without bespoke specialist equipment. Such measurements can be achieved using equipment that can be easily deployed in homes by study participants. Wider use of such methodology should be pursued to further understanding of human dose–response relationships with regards to the development of immediate IgE mediated allergy to airborne proteins and refinement of current risk assessment data. This paper also highlights limitations of current approaches such as dust sampling compared to inhalable protein measurement.
